# Recent advances in tuberculosis treatment: Towards shorter, safer, and more effective therapies

**DOI:** 10.1016/j.jctube.2026.100582

**Published:** 2026-01-14

**Authors:** Katherine Timboe, J.Brooks Jackson, Greta L. Becker

**Affiliations:** a Carver College of Medicine, University of Iowa, 451 Newton Rd, Iowa City, IA 52242, USA; b Department of Pathology, University of Iowa, 200 Hawkins Dr, Iowa City, IA 52242, USA; c Department of Internal Medicine, University of Iowa, 200 Hawkins Dr, Iowa City, IA 52242, USA

**Keywords:** Tuberculosis, drug-susceptible TB, drug-resistant TB, Treatment regimens, Host-directed therapy, latent TB

## Abstract

Tuberculosis (TB), caused by Mycobacterium tuberculosis, is the leading infectious cause of death globally. Although effective treatments are available, treatment length, drug toxicity, and the emergence of drug-resistant strains have challenged TB control efforts. Current clinical trials are focused on developing shorter, safer, and more effective regimens that incorporate both new and repurposed agents for the treatment of TB. This narrative review provides an overview of current and emerging treatment options for drug-susceptible, drug-resistant, and latent TB based on recent clinical trials and WHO guidelines.

## 1 History of tuberculosis and its treatment

Efforts to understand and treat tuberculosis (TB) have evolved over centuries, shaped by its long-standing impact on health and society. TB is an infectious disease caused by *Mycobacterium tuberculosis (M.Tb)*, that primarily affects the lungs (pulmonary TB) but can have widespread impact on other organ systems (extrapulmonary TB). TB has afflicted humans for thousands of years, with its existence described in writings of ancient cultures worldwide. TB has been known by many names – consumption, robber of youth, White Death – all descriptive of the ravaging effects TB has on the body.[1] Yet despite its impact, little was known as to how to treat the disease, and by the nineteenth century TB killed 1 of 7 people in the US and Europe; notable victims of TB include Frederic Chopin, Henry David Thoreau, John Keats, and US Presidents James Monroe and Andrew Jackson.[2], [3]

Robert Koch’s identification of *M.Tb* in 1882 opened the door for advances in the diagnosis and treatment of TB.[4] The development of the tuberculin skin test in 1908 allowed for quick diagnosis. Isolation in a sanatorium was widely used to treat and limit infection, and the BCG vaccine was developed in 1921 to prevent infection.[1] Yet it wasn’t until the discovery of antibiotics in the early twentieth century that TB treatment was revolutionized. Streptomycin and *para*-aminosalicylic acid (PAS), discovered in 1944, were the first drugs to demonstrate bacteriologic cure, marking a turning point in TB treatment.[5] These were soon followed by isoniazid in 1951, rifampin in 1957, and ethambutol in 1961; these drugs allowed for shorter treatment duration and became foundational drugs for the treatment of TB.[5] In 1993, the World Health Organization (WHO) introduced the Directly Observed Therapy Short-Course (DOTS) Strategy, which emphasized case detection and reporting, standardized and uninterrupted treatment, and a financial commitment by governments to sustain TB control activities, a program that is still in effect today.[6]

Despite these advances in therapeutic strategy, TB remains a major public health threat, with a quarter of the global population estimated to be infected with TB.[7] In 2023, an estimated 10.8 million people developed active disease, with the highest burden among low- and middle-income countries in Southeast Asia and Africa.[7] Multi drug-resistant/rifampin-resistant TB is estimated to account for 3.2% of all new cases.[7] TB remains the leading cause of death due to a single infectious agent, with 1.25 million deaths in 2023.[7] The WHO remains far from its goals of reducing TB incidence by 90% and TB deaths by 95% by 2035 compared to 2015 levels, as outlined in the endTB Strategy of 2015.[8] With these goals in mind, there remains a need to expand access to short, safe, and effective TB treatment regimens.

## 2 Drug-susceptible TB

### 2.1 Standard treatment

To better understand evolving therapies, we begin by reviewing treatments for drug-susceptible TB (DS-TB). DS-TB is defined as TB susceptible to both isoniazid and rifampin.[9] The WHO-recommended regimen for DS-TB involves a 6-month oral course of four drugs in two phases (2HRZE/4HR).[9] Phase 1, the intensive phase, consists of 2 months of daily isoniazid (H), rifampin (R), pyrazinamide (Z), and ethambutol (E).[9] Phase 2, the continuation phase, is 4 months of daily isoniazid and rifampin. Fixed-dose combination tablets are available and should be used when possible.[9] The recommendation for this regimen is based on a systematic review and *meta*-analysis of 312 arms of 57 different clinical trials dating back to 1965 and has been in practice since 2010.[9]

In contrast to adults, active TB in children is often non-severe and smear-negative. In 2018, the results of the SHINE trial demonstrated that in children and adolescents (3 months – 16 years) with non-severe TB, a shortened 4-month course of first line drugs (2HRZ(E)/2HR), was non-inferior to the 6-month standard treatment. The SHINE trial was key in identifying an affordable, safe, shortened treatment regimen for children with non-severe DS-TB without the need to incorporate newer drugs that have limited data on pediatric dosing and formulations.[10]

Each of these first line medications has a unique mechanism and side effect profile. Isoniazid exhibits bactericidal activity through inhibition of mycolic acid synthesis, an essential component of the mycobacterial cell wall, and is both selective and specific for *M.Tb*.[11] Isoniazid can cause hepatotoxicity, with 20% of patients developing mild liver injury and 1% developing severe hepatitis.[12] Overdoses of isoniazid (>30 mg/kg) can cause seizures, and other adverse effects include peripheral neuropathy, gastrointestinal effects, rash and pruritis.[12]

Rifampin exerts its action on *M.Tb* through inhibition of the bacterial DNA-dependent RNA polymerase.[13] Hepatotoxicity due to rifampin is uncommon, but well documented.[13] Major adverse effects are rare and are usually associated with intermittent administration.[14] Minor adverse effects include flushing, gastrointestinal effects, and an orange/red discoloration of the urine.[13] Rifampin is a strong inducer of several cytochrome P450 enzymes, and potential drug interactions should be considered.[15] Notably, rifampin can lead to subtherapeutic levels of certain anti-retroviral medications in patients with TB-HIV co-infection.[16]

Pyrazinamide is unique in its ability to target non-growing *M.Tb* persisters that are refractory to other drugs; this ability occurs via acidification of the bacterial cytoplasm and inhibition of membrane transport, protein synthesis, and RNA synthesis.[17] Therapies incorporating pyrazinamide are often associated with dose-dependent hepatotoxicity.[18] However, the exact role of pyrazinamide in liver injury is unclear since it is only used in combination with other anti-TB medications.[18] Other adverse effects of pyrazinamide include arthralgias, fatigue, gastrointestinal effects, rash and photosensitivity.[18].

Ethambutol is a bacteriostatic drug that interferes with arabinogalactan synthesis in the cell wall of multiplying mycobacterium.[19] Ethambutol is not typically associated with hepatotoxicity during TB treatment, although rare cases of liver injury have been reported.[20] Ethambutol can cause optic neuropathy which can present as decreased visual acuity, acquired color blindness, visual field defects, or blurred vision.[19] Other adverse effects include peripheral neuropathy and mental confusion.[19]

The 6-month 2HRZE/4HR standard regimen is highly effective, with favorable outcomes of at least 85% regularly reported to the WHO.[7] However, TB treatment continues to advance, and novel regimens are now emerging as effective alternatives for drug-susceptible TB.

### 2.2 Novel drug combinations and repurposed agents

Rifamycins, the class of antibiotics that include rifampin and rifapentine, are a cornerstone of TB treatment. Several recent studies have explored rifamycin dose adjustments or formulation changes to improve bactericidal activity and shorten TB treatment.

A recent phase III clinical trial, the RIFASHORT trial assessed two, 4-month regimens containing high-dose daily rifampin (1200 or 1800 mg [mg]) but failed to show non-inferiority to the standard 6-month regimen.[21] High dose rifampin was also evaluated in the TRUNCATE-TB trial, as described later in this article.[22]

Rifapentine (P) is a derivative of rifampin that exhibits a longer half-life, increasing the duration of exposure to the rifamycin.[23] A trial assessing a 4-month regimen incorporating intermittent rifapentine dosing in the continuation phase, the RIFAQUIN study, failed to show non-inferiority to standard treatment.[24] However, the TB Trials Consortium Study 31/AIDS Clinical Trials Group A5349 (Study 31/A5349), which assessed regimens incorporating daily rifapentine with or without moxifloxacin (M), showed that a 4-month, 2HPZM/2HPM regimen was non-inferior to a 6-month, 2HRZE/4HR regimen, with a similar safety profile and shorter time to stable sputum conversion.[23] The 2HPZM/2HPM regimen has now been approved by the WHO as an alternative to standard 6-month treatment in patients aged 12 and older with pulmonary DS-TB.[9]

Fluoroquinolones have also been an essential component of drug-resistant TB treatment for many years and are now being evaluated in several phase III clinical trials to shorten treatment duration for DS-TB. The REMox trial assessed two, 4-month moxifloxacin containing regimens, and the OFLOTUB study assessed a 4-month gatifloxicin containing regimen. Although these studies showed rapid initial decline in bacterial load, incorporation of a fluoroquinolone alone was not sufficient to show non-inferiority to the standard treatment.[25], [26] Study 31/A5349 marked a key breakthrough, where moxifloxacin combined with daily rifapentine in a 4-month regimen showed non-inferiority to the standard treatment.[23]

Although initially approved for treatment of drug-resistant TB, bedaquiline (B), pretomanid (Pa), delamanid (D) and linezolid (L) are now being tested for treatment of DS-TB. [27], [28], [29] A phase II clinical trial, SimpliciTB, tested a 4-month regimen of bedaquiline, pretomanid, moxifloxacin and pyrazinamide (BPaMZ) compared to standard treatment. The trial showed promise with faster sputum conversion in the BPaMZ group, but several hepatotoxic adverse events led to participant withdrawal, and the study was unable to show non-inferiority to standard treatment.[30] The TRUNCATE-TB trial, a phase II-III clinical trial, tested several regimens incorporating bedaquiline, linezolid, clofazimine (C), rifapentine, or high dose rifampin with standard first line drugs. Results showed that 8-week regimens containing bedaquiline and linezolid or high dose rifampin and linezolid showed non-inferiority to the standard treatment, representing the shortest effective treatment regimen for DS-TB.[22]

Some studies are evaluating the incorporation of new drugs into treatment regimens for DS-TB. The PanACEA-DECODE-01 and PanACEA-SUDOCU-01 studies recently evaluated 4-month regimens of bedaquiline, delamanid, moxifloxacin and a novel oxazolidinone, delpazolid or sutezolid, respectively, for treatment of DS-TB.[31], [32] Delpazolid was found to have an optimal dose of 1200 mg, and a maximum effect of sutezolid was not demonstrated in the dose range. Incorporation of both drugs demonstrated few oxazolidinone class toxicities.[31], [32] With further study, sutezolid and delpazolid have potential to be a safer, more effective alternative to linezolid in TB treatment regimens.

## 3 Drug-resistant TB

### 3.1 Isoniazid-resistant TB

Isoniazid-resistant TB (Hr-TB) is the most common form of drug-resistant TB, affecting approximately 8% of TB cases worldwide. Clinical trials in the 1970s and 1980s showed success with only the use of first-line therapies (rifampin, ethambutol, pyrazinamide) and these were long recommended by several international guidelines for treatment of Hr-TB.[33] However, more recent studies suggested that use of only first-line-therapies could result in poor outcomes for these patients.[33] In 2018, an individual patient data *meta*-analysis showed that addition of a fluoroquinolone to a 6-month regimen of (H)REZ was associated with significantly greater treatment success.[34] The WHO now recommends a 6-month treatment regimen of rifampin, ethambutol, pyrazinamide and levofloxacin (Lfx) for treatment of Hr-TB.[9] Although the addition of isoniazid has not been shown to improve treatment efficacy in this population, use of the HREZ fixed dose combination pill can be used for patient convenience.[9] Limitations of the (H)REZ-Lfx regimen include the need for drug-susceptibility testing (DST) to confirm rifampin susceptibility before initiating treatment, as well as the risk of excess hepatotoxicity with 6-month administration of pyrazinamide.[9]

### 3.2 Multidrug-resistant/rifampin-resistant TB

Multidrug-resistant TB (MDR-TB) is defined as TB resistant to both isoniazid and rifampin, while rifampin-resistant TB (RR-TB) is defined as TB resistant to rifampin but susceptible to isoniazid.[9] For many years, the standard treatment for MDR/RR-TB was a long-course, two-phase regimen, with the inclusion of second-line injectables such as kanamycin, capreomycin, or amikacin. The intensive phase consisted of 6-months of kanamycin, moxifloxacin, ethionamide, terizidone and pyrazinamide, followed by a 12–18 month continuation phase of all drugs except kanamycin.[35] In 2016, based on several observational studies from Bangladesh, Cameroon, and Niger, the WHO recommended a shorter 9–12-month injectable containing regimen for MDR/RR-TB.[36] This regimen consisted of a 4–6 month intensive phase (kanamycin, gatifloxacin, prothionamide, clofazimine, high-dose isoniazid, pyrazinamide, ethambutol), followed by a 5-month continuation phase (gatifloxacin, clofazimine, pyrazinamide, ethambutol).[36] However, the use of injectables carries a serious risk of adverse effects, most notably ototoxicity.[37]

In 2018, the WHO issued an urgent update to its TB treatment guidelines. The update emphasized the feasibility of a longer, fully oral treatment regimen for MDR-TB, although optimization of this regimen was ongoing. The update also encouraged discontinuation of kanamycin and capreomycin in all MDR-TB regimens due to increased risk of treatment failure/relapse. If an injectable agent was needed to be incorporated into the regimen, amikacin (or streptomycin) was recommended.[38]

In 2019, an optimized 12–18 month fully oral regimen was recommended by the WHO for treatment of MDR-TB. This regimen included the use of at least 4 medications, chosen sequentially from 3 groups. As many agents as possible from Group A (levofloxacin/moxifloxacin, bedaquiline, linezolid) were to be used, followed by at least one agent from Group B (clofazimine, cycloserine/terizidone), and supplemented with agents from Group C (ethambutol, delamanid, pyrazinamide, imipenem-cilastatin/meripenem, amikacin, ethionamide/prothionamide, p-amino-salicylic acid) as needed. Of note, ethionamide/prothionamide and p-amino-salicylic acid were only to be used if incorporation of bedaquiline, linezolid, clofazimine, or delamanid was not possible.[39]

In 2020, the WHO recommended the use of a standard, 7-drug, 9-month, all-oral regimen for patients with MDR/RR-TB.[40] The regimen consists of 4 months of ethionamide and high-dose isoniazid, 6 months of bedaquiline, and 9 months of levofloxacin/moxifloxacin, ethambutol, pyrazinamide and clofazimine, with the option to substitute 2-months of linezolid for ethionamide.[40] This recommendation followed an analysis of programmatic data from the use of a 9-month bedaquiline-containing regimen in South Africa, which showed higher treatment success rates and less loss to follow up than a 9–12-month injectable containing regimen.[40] However, this recommendation was based on “very low” certainty of evidence due to potential risk of bias and lack of data on adverse events; more evidence on shorter, all-oral regimens was needed.[40]

In 2022, evidence from a phase 3 randomized clinical trial, STREAM 2, demonstrated non-inferiority and reduced risk of ototoxicity of two bedaquiline-containing regimens when compared to a 9-month injectable-containing regimen (moxifloxacin, clofazimine, ethambutol, pyrazinamide with a 4-month intensive phase of isoniazid, prothionamide and kanamycin) whose efficacy was demonstrated in the STREAM 1 trial. Effective regimens in STEAM 2 included a 9-month all-oral regimen (moxifloxacin, clofazimine, ethambutol, pyrazinamide, and bedaquiline with a 4-month intensive phase of high-dose isoniazid and prothionamide) and a 6-month injectable-containing regimen (levofloxacin, clofazimine, pyrazinamide, bedaquilline with a 2-month intensive phase of isoniazid and kanamycin). [41]

Later that year, results of the TB-PRACTECAL study demonstrated non-inferiority of a 6-month all-oral regimen of B, Pa, L, and M (BPaLM) to the 9–20-month local standard of care for RR-TB.[42] Unfavorable outcomes (death, treatment failure, treatment discontinuation, loss to follow-up, TB recurrence) were reported in only 11% of patients on BPaLM, compared to 48% of those receiving the standard of care.[42] Safety outcomes were also improved in the BPaLM group, with lower percentages of total adverse events, hepatic disorders, QTcF prolongation and peripheral neuropathy compared to standard of care.[42] Recruitment for the TB-PRACTECAL study was terminated early for efficacy.[42]

A notable limitation of the BPaLM regimen in patients with MDR/RR-TB is the inclusion of pretomanid, which is not recommended for patients who are pregnant, breastfeeding, or under age 14.[9] In 2025, the endTB trials showed non-inferiority of three, 9-month, all-oral regimens (BLMZ, BCLLfxZ, BDLLfxZ) compared to an individualized 18–24 month standard treatment, with favorable outcomes in 85% of patients with RR-TB.[43] All three of these regimens contain drugs with formulations for children and are safe in pregnant persons.[43] In preprint, the results of the BEAT TB trial in South Africa by Conradie et al have expanded on the endTB trials, demonstrating non-inferiority of a 6-month BDCLLfx regimen to a standard 9-month linezolid containing regimen.[44]

The WHO treatment guidelines for MDR/RR-TB now recommend the use of the 6-month BPaLM regimen in non-pregnant persons over 14 or the use of the 6-month BDCLLfx regimen for persons of all ages, including those who are pregnant or breastfeeding.[9] The use of clofazimine can be dropped from the BDCLLfx regimen when fluoroquinolone susceptibility is confirmed.[9] In patients who are ineligible for the 6-month regimens, the standard 9-month all-oral regimen or modified 9-month all-oral regimens (BLMZ, BCLLfxZ, BDLLfxZ) can be used.[9] Due to the inclusion of individuals with significant comorbidities and extensive disease in the endTB trials in which they were tested, the modified 9-month regimens are preferable in these populations.[9], [44] Among modified regimens, BLMZ is recommended over BCLLfxZ, and BCLLfxZ over BDLLfxZ.[9]

### 3.3 Pre-extensively drug-resistant TB and extensively-drug resistant TB

Pre-extensively drug-resistant TB (pre-XDR-TB) is defined as TB resistant to rifampin and at least one fluoroquinolone.[9] Standard treatment for pre-XDR has previously involved long, individualized regimens containing three group A agents and at least one Group B agent.[9]

A 6–9-month regimen of bedaquiline, delamanid, linezolid, and clofazimine (BDLC) was evaluated in pre-XDR/XDR-TB patients during the BEAT-India study.[45] Favorable outcomes were achieved in 91% of pre-XDR-TB patients, although adverse effects due to linezolid and clofazimine were considerable.[45] A limitation of the BEAT-India study was its lack of a comparator control group. The end-TB-Q trial addressed this limitation in the first phase III randomized control trial to evaluate a BDLC regimen exclusively in pre-XDR-TB patients. End-TB-Q examined a tailored strategy for treatment of pre-XDR-TB based on extent of disease; patients with less extensive disease received a 6-month regimen, while patients with more extensive disease received 9-months.[46] Favorable outcomes were achieved in 87% of patients on BDLC compared to 89% of patients on a conventional long regimen, and the regimen was more effective (93%) in patients with less extensive disease.[46] Results of the end-TB-Q trial suggest that a shorter BDLC regimen may be a good option for pre-XDR-TB patients with less extensive disease, but insufficient for those with severe disease.[46] The WHO now recommends the BPaL regimen for treatment of pre-XDR TB in non-pregnant patients over age 14 and the BDLC regimen for children and pregnant persons.[9] Further investigation into tailoring these therapies based on disease severity is needed.

Prior to 2021, XDR-TB was defined as TB resistant to rifampin, a fluoroquinolone, and a second line injectable agent. By incorporating newer TB drugs, such as bedaquiline, several studies demonstrated efficacy of shorter, all-oral regimens in this XDR-TB population. In 2020, the Nix-TB trial evaluated a 6–9-month regimen of bedaquiline, pretomanid, and 1200 mg daily linezolid (BPaL) in patients with XDR-TB or treatment-refractory MDR-TB.[47] Ninety percent of patients had favorable outcomes, but the regimen was limited by adverse events due to linezolid toxicity, including peripheral neuropathy, myelosuppression, and anemia.[47] The promising results of the Nix-TB trial paved the way for the ZeNix trial in 2022, which assessed lower linezolid doses in XDR-TB and found that 600 mg daily for 6 months maintained efficacy while reducing adverse events.[48] That same year, the results of the BEAT-India study demonstrated success of a 6–9-month BDLC regimen, with 69% of XDR-TB patients achieving a favorable outcome.

In 2021, the WHO revised its definition of XDR-TB to reflect reduced reliance on injectable agents due to growing concern of resistance to newer drugs, with increasing reports of bedaquiline resistance, particularly in countries with limited access to genotypic DST. XDR-TB is now defined as TB resistant to rifampin, a fluoroquinolone, and one additional Group A agent (bedaquiline or linezolid).[49] There is not significant evidence to support shorter regimens for patients with resistance to these drugs. Current WHO recommendations reserve the use of longer, 18–20-month regimens, for these cases of XDR-TB. Longer regimens must be individualized to the patient’s resistance profile and should include at least 4 agents likely to be effective. As many Group A agents as possible should be used, followed by Group B agents, and then Group C agents as necessary. If using an injectable agent (amikacin/streptomycin), the intensive phase should be at least 6 months. Continuation of treatment 15–17 months after sputum conversion is recommended in these patients.[9]

A summary of WHO treatment guidelines for active tuberculosis is listed in Table 1.

**Table 1:** Summary of WHO Treatment Guidelines for Active Tuberculosis.

**TB Susceptibility**	**Drug Regimen**	**Duration**	**Abbreviation**	**Considerations**
DS-TB	Isoniazid, rifampin, pyrazinamide, ethambutol	6 months	2HRZE/4HR	Children and adults
DS-TB	Isoniazid, rifapentine, pyrazinamide, moxifloxacin	4 months	2HPZM/2HPM	Patients aged 12 years and older
DS-TB	Isoniazid, rifampin, pyrazinamide, (ethambutol)	4 months	2HRZ(E)/2HR	Patients between 3mo and 16 years of age with non-severe TB
Hr-TB	(Isoniazid), rifampin, pyrazinamide, ethambutol, levofloxacin	6 months	6(H)RZE-Lfx	Children and adults
MDR/RR-TB	Bedaquiline, pretomanid, linezolid, moxifloxacin	6 months	BPaLM	Non-pregnant patients aged 14 years and older
MDR/RR-TB	Bedaquiline, delamanid, linezolid, levofloxacin	6 months	BDLLfx	Children and adults, including pregnant persons and PLWH
MDR/RR-TB	Bedaquiline, levofloxacin/moxifloxacin, ethionamide/linezolid, ethambutol, isoniazid	9 months	−	Children and adults, including PLWH. Safe in pregnant persons if linezolid is used
MDR/RR-TB	Bedaquiline, linezolid, moxifloxacin, pyrazinamide	9 months	BLMZ	Children and adults, including pregnant persons and PLWH, not eligible for 6-month regimen
MDR/RR-LLfxCZTB	Bedaquiline, linezolid, levofloxacin, clofazimine, pyrazinamide	9 months	BLLfxCZ	Children and adults, including pregnant persons and PLWH, not eligible for 6-month regimen
MDR/RR-TB	Bedaquiline, delamanid, linezolid, levofloxacin, pyrazinamide	9 months	BDLLfxZ	Children and adults, including pregnant persons and PLWH, not eligible for 6-month regimen
Pre-XDR TB	Bedaquiline, pretomanid, linezolid	6 months	BPaL	Non-pregnant patients aged 14 years and older
Pre-XDR TB	Bedaquiline, delamanid, clofazimine, linezolid,	6 months	BDCL	Children and adults, including pregnant persons and PLWH
MDR-RR TB, pre-XDR TB, XDR-TB	Levofloxacin/moxifloxacin, bedaquiline, linezolid and one Group B agent (clofazimine, cycloserine, terizidone)	18–20 months	−	Children and adults

## 4 Latent TB infection

Latent TB infection (LTBI) is defined as infection causing an immunologic response to *M.Tb* antigens, as evidenced by a TST or interferon-gamma release assay, without clinical signs and symptoms of TB.[50] Individuals with LTBI cannot transmit disease but are at a 5–10% risk of developing active TB in their lifetime.[50] In deciding who should be tested and treated for LTBI, the WHO adheres to the principle that individual benefit should outweigh risk.[50] At risk populations who may benefit from LTBI testing and treatment include people living with HIV (PLWH), household contacts of people with active TB, patients initiating anti-TNF treatment, patients on dialysis, patients preparing for organ/hematological transplant, patients with silicosis, incarcerated persons, healthcare workers, persons experiencing homelessness, persons with illicit drug use, and immigrants from countries with high incidence of TB.[50]

Conventional therapy for latent TB is isoniazid monotherapy, with 6–9-month regimens of isoniazid (6H/9H) demonstrating efficacies of 60–90%.[51] However, isoniazid hepatotoxicity is a common complication of treatment for latent TB and can be severe.[52] Several studies have investigated alternative options for the treatment of latent TB. A 4-month regimen of daily rifampin (4R) and a 3-month regimen of weekly rifapentine and isoniazid (3HP) have shown non-inferiority to 9H, with increased adherence and decreased hepatotoxic events.[53], [54] The 4R regimen presents a strong option if exposure to Hr-TB is known. A 3-month regimen of daily isoniazid and rifampin (3HR) has shown good efficacy and adherence, but has not been directly compared to 9H in clinical trials.[51] In countries with low TB incidence the 9H, 4R, 3HP and 3HR regimens are recommended as alternatives to 6H for treatment of LTBI in patients exposed to DS/Hr-TB.[50] In countries with high TB incidence, the 3HP regimen is recommended for adults and the 3HP or 3HR regimens are recommended for children.[50]

With the growing incidence of MDR/RR-TB, a strategy for preventative treatment in close contacts of these patients is needed. In 2024, two trials, TB-CHAMP and V-QUIN, assessed the use of a 6-month levofloxacin regimen (6Lfx) in close contacts of patients with MDR/RR-TB.[55], [56] TB-CHAMP showed a 56% reduction in TB incidence in MDR/RR-TB exposed children when compared to placebo, with V-QUIN demonstrating a 45% reduction in TB incidence in MDR/RR-TB exposed adolescents/adults when compared to placebo.[55], [56] Although the results of these trials were not statistically significant, the WHO now recommends the 6Lfx regimen for preventative treatment of MDR/RR-TB close contacts of all ages.[55], [56], [57]

## 5 Host-directed therapies

Host-directed therapies (HDT) are an emerging field in adjuvant therapy for TB. HDT aims to improve patient outcomes by enhancing the host immune response and reducing tissue damage that occurs from excessive inflammation.[58] This strategy is accomplished by repurposing drugs used for non-infectious diseases such as cancer, diabetes, and cardiovascular disease.[59] The development of HDTs is particularly important in the response to drug-resistant TB, as these therapies do not depend on typical anti-microbial mechanisms for their action.[59]

Statins, such as atorvastatin and rosuvastatin, show immunoregulatory effects through enhancement of cell autophagy and macrophage maturation.[58] However, clinical trials testing the effects of statins on TB treatment have been inconclusive. A phase II study of adjuvant atorvastatin demonstrating improved microbiological outcomes, while a phase II study of adjuvant rosuvastatin showed no significant benefit.[60], [61] Everolimus, an mTOR inhibitor, also enhances autophagy.[62] A phase II clinical trial has shown the potential of adjuvant everolimus to enhance FEV_1_ recovery after TB treatment.[63] Metformin, a biguanide used for treatment of type II diabetes mellitus, enhances autophagy through indirect mTOR inhibition.[58] In clinical trials, adjuvant metformin demonstrated no effect on time to sputum conversion, but was associated with improved radiographical changes and decreased inflammatory markers.[64] Vitamin D enhances immune function by promoting production of anti-microbial peptides and modulating excess inflammation.[65] A recent *meta*-analysis of 26 randomized control trials found that vitamin D supplementation did not reduce time to sputum conversion, but was associated with a lower TB score, indicating reduced symptom-burden.[65]

In addition to those described above, several other therapies have shown promising immunomodulatory and anti-inflammatory effects for treatment of *M.Tb* in preclinical models, but have not yet been tested in a clinical setting.[58], [59] As it stands, no HDTs are currently widely used in the treatment of TB, as extensive clinical evidence is lacking. More research is needed on the effects these adjuvant therapies may have on TB treatment efficacy and safety outcomes.

## 6 Investigational medicines in the TB pipeline

TB treatment regimens are rapidly evolving, with several promising new compounds in development for both DS-TB and DR-TB. Two agents, quabodepistat (OPC-167832) and BTZ-043, target the *DprE1*enzyme, essential for *M.Tb* cell wall synthesis. Quabodepistat, in combination with bedaquiline and delamanid, was recently tested in a multicenter, Phase IIb/c clinical trial, though results are not yet published. BTZ-043 is currently undergoing dose optimization in the Phase IIb DECISION trial.[66]

Alpibectir, a novel potentiator of ethionamide activity was studied in the TASK-010 trial, with further evaluation in the UNITE4TB Program’s, ENABLE study, where its efficacy is being assessed with first-line agents. TBAJ876, a next generation diarylquinoline related to bedaquiline, is being tested in combination with pretomanid and linezolid in a Phase II trial against the standard HRZE regimen. Sudapyridine, another diarylquinoline analogue, is being evaluated in the Phase III, WISH trial as an alternative to bedaquiline.[66]

## 7 Barriers to access and implementation

For new TB treatment regimens to be effective outside of the research sphere, they need to be programmatically introduced in target nations. The first step is to establish a national expert committee/technical working group (TWG) to assist healthcare providers in coordinating the necessary changes for introduction of a new regimen. The TWG conducts a needs-based assessment for the introduction of the new regimen and identifies an initial adopting site. A plan for access to diagnostics, drugs, and consumables for the introductory site needs is developed, and national policy documents, clinical guidelines, and standard operating procedures are updated to reflect incorporation of the new regimen. The introductory site needs to be closely monitored to identify patient outcomes, adherence to protocols, and the costs/resources needed for implementation. Taking this data into account, budgets, supply chains, policies and protocols are usually updated for nationwide scale-up.[9]

Reducing the global TB burden will require addressing barriers to access and implementation of new treatment strategies. With 99% of new TB cases occurring in low- and middle-income countries, the cost of TB treatment is a major concern.[7] Drug prices for a 2HRZE/4HR regimen for DS-TB are approximately US$46, while a full course of BPaLM for RR-TB costs approximately US$432.[67] One hundred twenty-two countries provided free access to TB treatment in 2024, with countries such as Uganda reporting significant improvements in treatment success with this policy.[7], [68] However, about 50% of households still face catastrophic costs, direct or indirect, during TB diagnosis and treatment.[7] As funding for TB prevention, diagnostics and treatment continues to fall, the need for affordable options has become more pressing.[7] Based on a modeling analysis of the economic implications of novel treatment regimens, regimens that shorten duration and improve adherence can yield considerable patient savings.[67] Incorporation of newer, more expensive drugs may be economically feasible if they are more effective and can limit long term retreatment, recurrence, or disability costs.[67] As new treatment regimens become available, it is important to consider the cost of their implementation.

Limited access to diagnostic testing is another major hurdle to TB treatment implementation. Some gaps in the diagnostic cascade include failure to access diagnostic facilities, failure to receive a complete diagnosis despite reaching a health facility, and failure to seek care despite access to facilities.[69] Rapid identification of active TB and its drug susceptibility are essential to halt the transmission cascade. Culture methods are the gold-standard for diagnosis of TB but has a long turnaround time and requires significant infrastructure.[70] Rapid molecular diagnostic tests, such as the Xpert MTB/RIF have become the most commonly used tests worldwide.[70] However, these tests fail to identify other forms of drug resistance, and their expense limits use in low-income settings.[70] In order to properly implement new treatments for DS and DR-TB, it is necessary to address these gaps in availability of diagnostic and drug-susceptibility testing.

## 8 Priorities for future research

A continued search for short, effective, and safe treatments is a priority. Many of the recommended regimens, especially for DR-TB, could benefit from additional research into optimal dosing and duration. Exploration of other combination regimens that may have better side-effect profiles, such as those eliminating linezolid, would be beneficial. Safety and efficacy of newer treatment regimens within special populations, such as children, pregnant persons, PLWH and patients with extra-pulmonary disease, is especially needed, as these populations are often excluded from clinical trials and extensive research is lacking.[9] Furthermore, second-line drugs, such as bedaquiline and linezolid, are increasingly being incorporated into regimens for the treatment of DS-TB in order to shorten treatment time and improve tolerability. Widespread use of second-line agents in DS-TB may contribute to resistance amplification. Continued research into these potential risks is needed to help safeguard the effectiveness of these drugs against DR-TB strains, where use of second-line agents is necessary to achieve sputum conversion.

Funding.

This work was supported by the Mark Gilbert and Karen Simmonds Research Gift.

## CRediT authorship contribution statement

**Katherine Timboe:** Writing – original draft. **J.Brooks Jackson:** Writing – review & editing, Conceptualization. **Greta L. Becker:** Writing – review & editing, Conceptualization.

## Declaration of competing interest

The authors declare that they have no known competing financial interests or personal relationships that could have appeared to influence the work reported in this paper.
